# Increase in resistance to anticancer drugs involves occludin in spheroid culture model of lung adenocarcinoma A549 cells

**DOI:** 10.1038/s41598-018-33566-w

**Published:** 2018-10-11

**Authors:** Hiroaki Eguchi, Risa Akizuki, Ryohei Maruhashi, Mitsutoshi Tsukimoto, Takumi Furuta, Toshiyuki Matsunaga, Satoshi Endo, Akira Ikari

**Affiliations:** 1 From the Laboratory of Biochemistry, Department of Biopharmaceutical Sciences, Gifu Pharmaceutical University, Gifu, Japan; 2 Department of Radiation Biosciences, Faculty of Pharmaceutical Sciences, Tokyo University of Science, Chiba, Japan; 3 Department of Pharmaceutical Chemistry, Kyoto Pharmaceutical University, Kyoto, Japan

## Abstract

Chemoresistance is a serious issue in the therapy of many cancers, but the molecular mechanism is little understood. The mRNA level of occludin (OCLN), a tight junctional protein, was increased in the cisplatin (CDDP), doxorubicin (DXR), 7-ethyl-10-hydroxy-camptothecin, or gemcitabine-resistant human lung adenocarcinoma A549 cells. Here, we investigated the regulatory mechanism and pathophysiological role of OCLN. OCLN was mainly localized at tight junctions in A549 and CDDP-resistant A549 (A549/CDDP) cells. The level of p-Akt in A549/CDDP cells was higher than that in A549 cells, and the mRNA and protein levels of OCLN were suppressed by a phosphoinositide 3-kinase (PI3K)/Akt pathway inhibitor, LY-294002, suggesting that a PI3K/Akt pathway is involved in the elevation of OCLN expression. The overexpression of OCLN in A549 cells decreased paracellular permeability to DXR. Cytotoxicity to CDDP was unaffected by OCLN-overexpression in 2D culture model. In 3D culture model, the spheroid size, hypoxic level, and cell viability were significantly elevated by CDDP resistance, but not by OCLN-overexpression. The accumulation inside the spheroids and toxicity of DXR were correlated with OCLN expression. Our data suggest that OCLN is not directly involved in the chemoresistance, but it enhances chemoresistance mediated by suppression of accumulation of anticancer drugs inside the spheroids.

## Introduction

The pathology of lung cancer can be divided into non-small cell lung cancer (NSCLC) and small cell lung cancer. NSCLC accounts for approximately 80% of lung cancers diagnosed worldwide and contributes to poor survival^1^. NSCLC is classified as adenocarcinoma, squamous cell carcinoma, and large cell carcinoma. Among them, adenocarcinoma is the most popular type and shows little sensitivity to chemotherapy. Cisplatin (CDDP) is a platinum-based drug that is widely used in lung cancer treatment, but its effectiveness significantly decreases after the development of CDDP resistance. An acquired drug resistance can confer cross-resistance to diverse anticancer drugs, thereby causing inefficient treatment. Over 50% of patients undergoing lung cancer surgery acquire a chemoresistant phenotype^2^. Multiple mechanisms including induction of drug efflux pumps, anti-apoptosis factors, and drug-metabolizing enzymes are involved in the development of drug resistance^3^. The formation of tumor microenvironment is also involved in the development of chemoresistance^4^, but the molecular mechanism remain elusive.

Both malignant and non-malignant cells formed the tumor microenvironment *in vivo* during developing tumors. The inside cells of microenvironment experience severe stress conditions including hypoxia, oxidative stress, and so on^5^. Hypoxic stress causes adaptive responses such as the induction of genes transcription implicated in chemoresistance. A spheroid is a three-dimensional (3D) *in vitro* tumor model and resembles *in vivo* situation^6^. Cancer cells in 3D spheroid cultures often represent greater resistance to anticancer drugs than the cells grown in 2D monolayer cultures^7^. However, the molecular mechanisms of chemoresistance are not entirely elucidated in 3D culture model. We recently reported that claudin-1 (CLDN1) and CLDN2, components of tight junctions (TJs), decrease chemosensitivity to doxorubicin (DXR) in 3D-cultured A549 cells established from human lung adenocarcinoma^8,9^.

TJs regulate not only paracellular solute and ion transports, but also restrict the diffusion of membrane components^10–12^. In addition, TJs are involved in the coordination of cell differentiation, proliferation, and migration. Transmembrane proteins including occludin (OCLN), CLDNs, and junctional adhesion molecule exist in the bicellular TJs^13,14^. Tricellulin exists in the tricellular TJs of neighboring cells^15^. These proteins are scaffolded by zonula occludens (ZO)-1 that interacts with the actin cytoskeleton. CLDNs constitute a family with at least 24 different members in human and these subtypes can form homo- or heterophilic interactions between adjacent cells^16,17^. In contrast, OCLN is the first identified integral membrane protein of TJs and has no subtype^18^. In the respiratory system, OCLN is expressed in bronchial airway and alveolar cells under physiological conditions^19,20^. In an immunohistochemical analysis, OCLN is expressed in human lung adenocarcinomas, but not in squamous cell carcinomas and large cell carcinomas^21^. In addition, the mRNA level of OCLN is increased in adenocarcinomas compared to normal lung tissue^22^. However, the pathophysiological role of OCLN in lung adenocarcinoma tissue has not been clarified yet.

The expression level of OCLN in CDDP-resistant A549 (A549/CDDP) cells was higher than that in parent A549 cells. Therefore, we investigated the regulatory mechanism and pathophysiological role of OCLN expression. The elevation of mRNA and protein levels of OCLN was inhibited by a phosphoinositide 3-kinase (PI3K) inhibitor, LY-294002, in A549/CDDP cells. Cytotoxicity to DXR was not changed by OCLN-overexpression in 2D culture model, but paracellular permeability to DXR was decreased. Additionally, OCLN overexpression decreased the accumulation and cytotoxicity of DXR in 3D culture model. These results indicate that OCLN may be implicated in the promotion of chemoresistance in A549 spheroid cells.

## Results

### Effect of resistance to anticancer drugs on the expression and localization of OCLN in A549 cells

**Figure 1:**
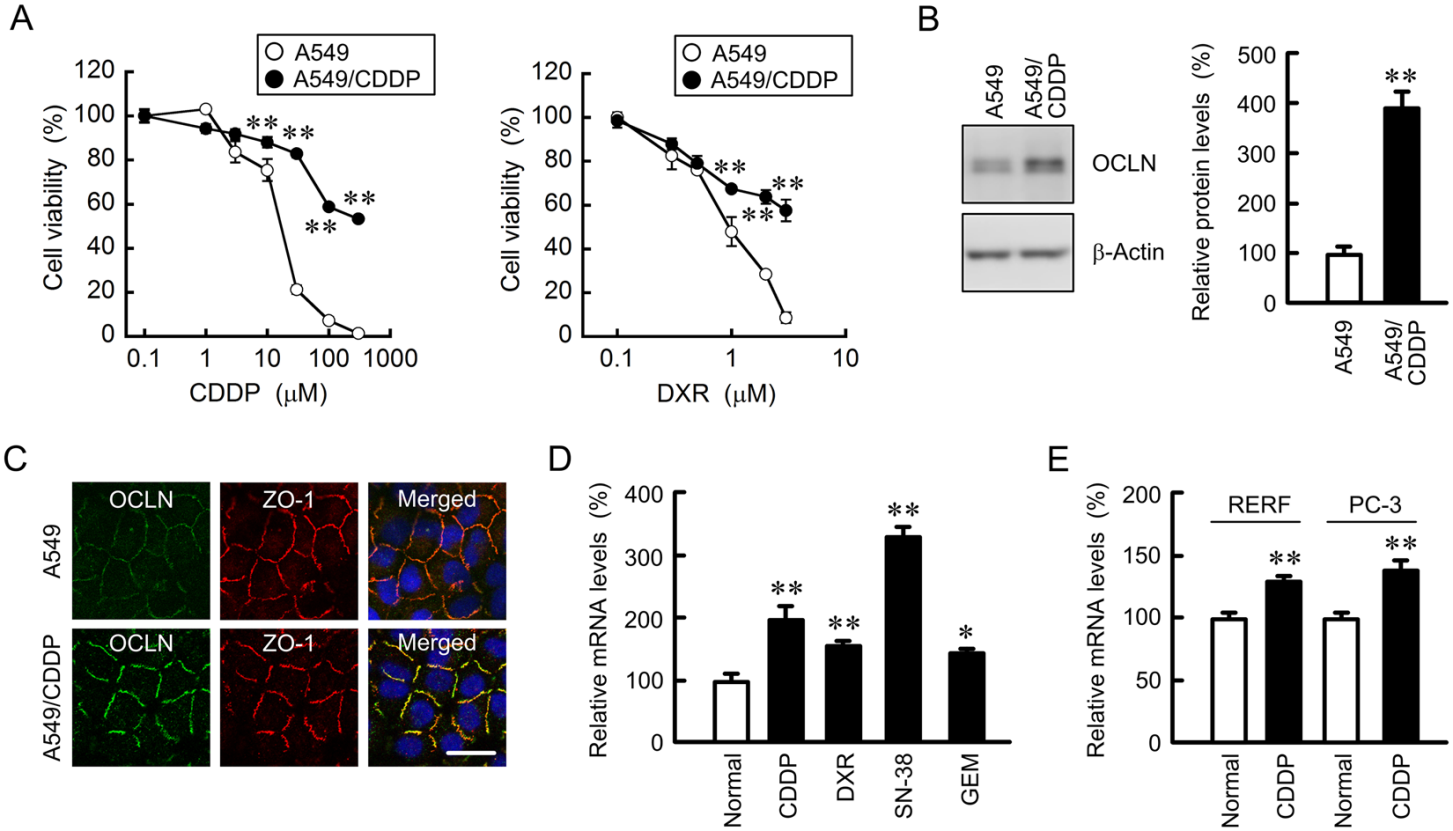
Elevation of mRNA level of *OCLN* in chemoresistant A549 cells. (**A**) A549 and A549/CDDP cells were cultured on a 96 well plate for 48 h followed by incubation with CDDP or DXR for additional 48 h at the concentrations indicated. Cell viability was measured by WST-1 assays. (**B**) The protein levels of OCLN and β-actin in cell lysates were examined by western blotting and shown as a percentage of the value in A549 cells. The full-length blot images are shown in Supplementary Fig. S1. (**C**) Immunofluorescence stainings with anti-OCLN (green) and anti-ZO-1 (red) antibodies were performed. The right images show the merged picture with DAPI (blue). Scale bar represents 10 µm. (**D**) The expression levels of OCLN mRNA in anticancer drug-resistant cells are shown as a percentage of the values in parent cells (normal). (**E**) Total RNA was isolated from normal or CDDP-resistant RERF-LC-MS (RERF) and PC-3 cells. The expression levels of OCLN mRNA are shown as a percentage of the values in normal. n = 3–4. **P < 0.01 and *P < 0.05 compared with A549 or normal. NS, *P* > 0.05.

CDDP, an anticancer drug containing platinum, concentration-dependently increased toxicity of A549 cells (Fig. 1A). Compared with the parent cells, the chemosensitivity to CDDP was significantly lower at above 10 μM in A549/CDDP cells. In addition, the sensitivity to DXR was also attenuated by developing the CDDP resistance, indicating that A549/CDDP cells acquired cross resistance to DXR. The protein level of OCLN in A549/CDDP cells was significantly higher than that in A549 cells (Fig. 1B). Immunofluorescence measurements showed that OCLN was mainly colocalized with ZO-1 and DAPI, indicating that OCLN are distributed in the TJs (Fig. 1C). The CDDP resistance increased the fluorescence intensity of OCLN at the TJs in A549/CDDP cells, but it did not change the intracellular localization of OCLN. The mRNA level of *OCLN* in A549/CDDP cells is higher than that in normal cells (Fig. 1D), indicating that CDDP resistance increases OCLN expression at the transcriptional step. Similarly, the mRNA level of *OCLN* was increased by resistance to DXR, gemcitabine (GEM), and 7-ethyl-10-hydroxy-camptothecin (SN-38), a metabolite of the camptothecin derivative CPT-11, in A549 cells. In addition, that of *OCLN* was increased by resistance to CDDP in human lung adenocarcinoma RERF-LC-MS and PC-3 cells (Fig. 1E). These results indicated that the resistance to anticancer drugs increased the expression and tight junctional localization of OCLN in A549 cells.

### Elevation of OCLN expression by phosphoinositide 3-kinase (PI3K)/Akt signaling pathway in A549/CDDP cells

**Figure 2:**
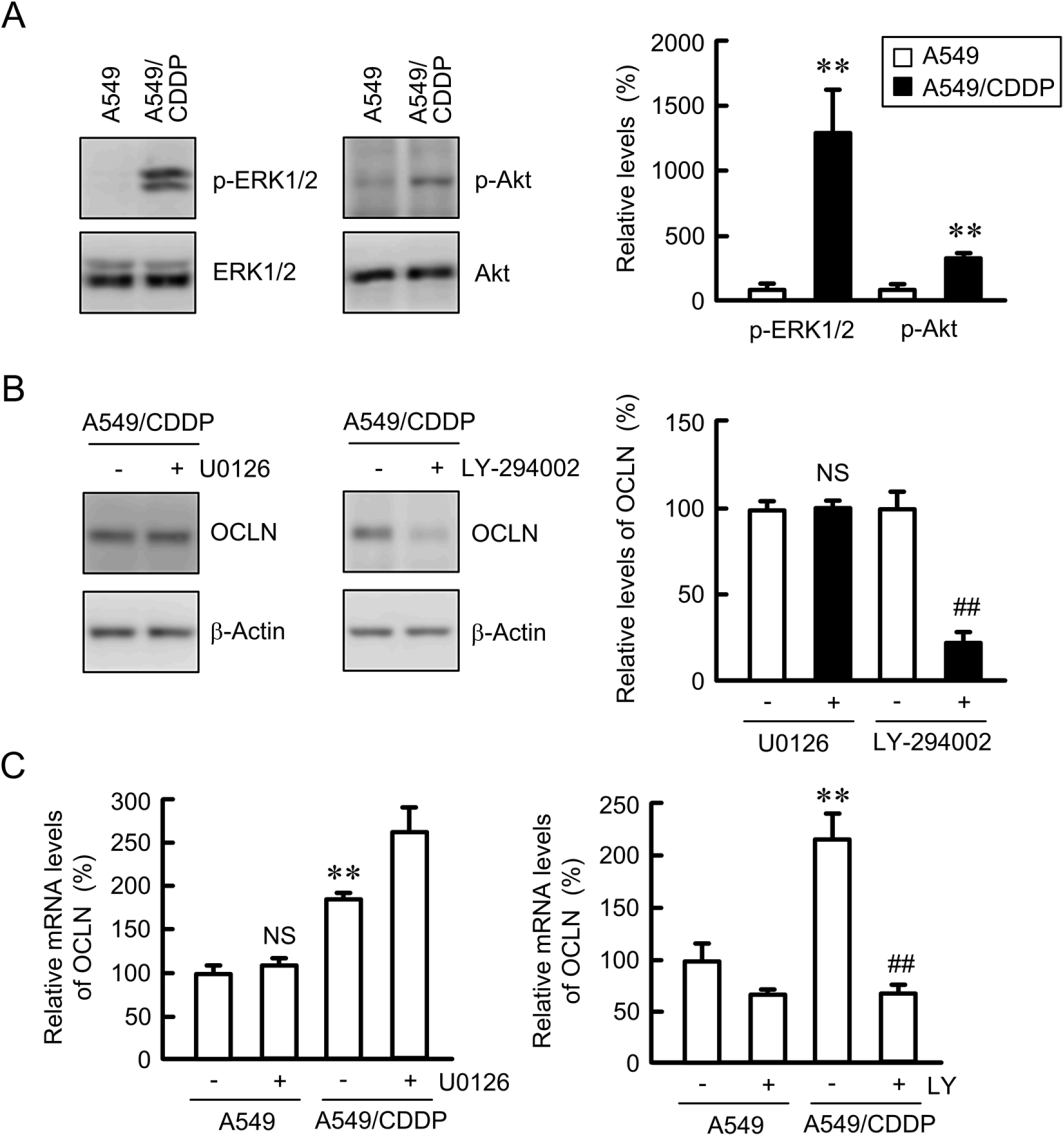
Decrease in OCLN expression by LY-294002 in A549/CDDP cells. (**A**) The expression of p-ERL1/2, ERK1/2, p-Akt, and Akt in whole cell extracts was examined by western blotting. The levels of p-ERK1/2 and p-Akt are shown as a percentage of the values in A549 cells. The full-length blot images are shown in Supplementary Fig. S2A. (**B**) A549/CDDP cells were treated with or without 10 μM U0126 and 10 µM LY-294002 for 24 h. The expression of OCLN and β-actin in cell lysates was examined by western blotting. The protein levels of OCLN are shown as a percentage of the values in the absence of inhibitor. The full-length blot images are shown in Supplementary Fig. S2B. (**C**) A549 and A549/CDDP cells were treated with or without U0126 and LY-294002 for 6 h. The expression levels of OCLN mRNA are shown as a percentage of the values in the absence of inhibitor. n = 3–4. **P < 0.01 compared with A549. ^##^*P* < 0.01 compared with –LY. NS, *P* > 0.05 compared with – U0126.

To clarify the mechanism of OCLN upregulation by CDDP resistance, we investigated the involvement of mitogen-activated protein kinase kinase (MEK)/ extracellular signal-regulated kinase (ERK) and PI3K/Akt signaling pathways. The p-ERK1/2 and p-Akt levels in A549/CDDP cells were significantly higher than those in the parent cells (Fig. 2A). In contrast, there is no difference in the total levels of ERK1/2 and Akt. The protein level of OCLN was suppressed by LY-294002 in A549/CDDP cells, but not by U0126, a MEK/ERK pathway inhibitor (Fig. 2B). Similarly, the CDDP resistance-induced elevation of OCLN mRNA was suppressed by LY-294002 (Fig. 2C). In contrast, U0126 increased the level of OCLN mRNA in A549/CDDP cells. These results indicated that PI3K/Akt pathway may be involved in the elevation of OCLN expression in A549/CDDP cells.

### Effects of CDDP resistance and OCLN overexpression on paracellular permeability

**Figure 3:**
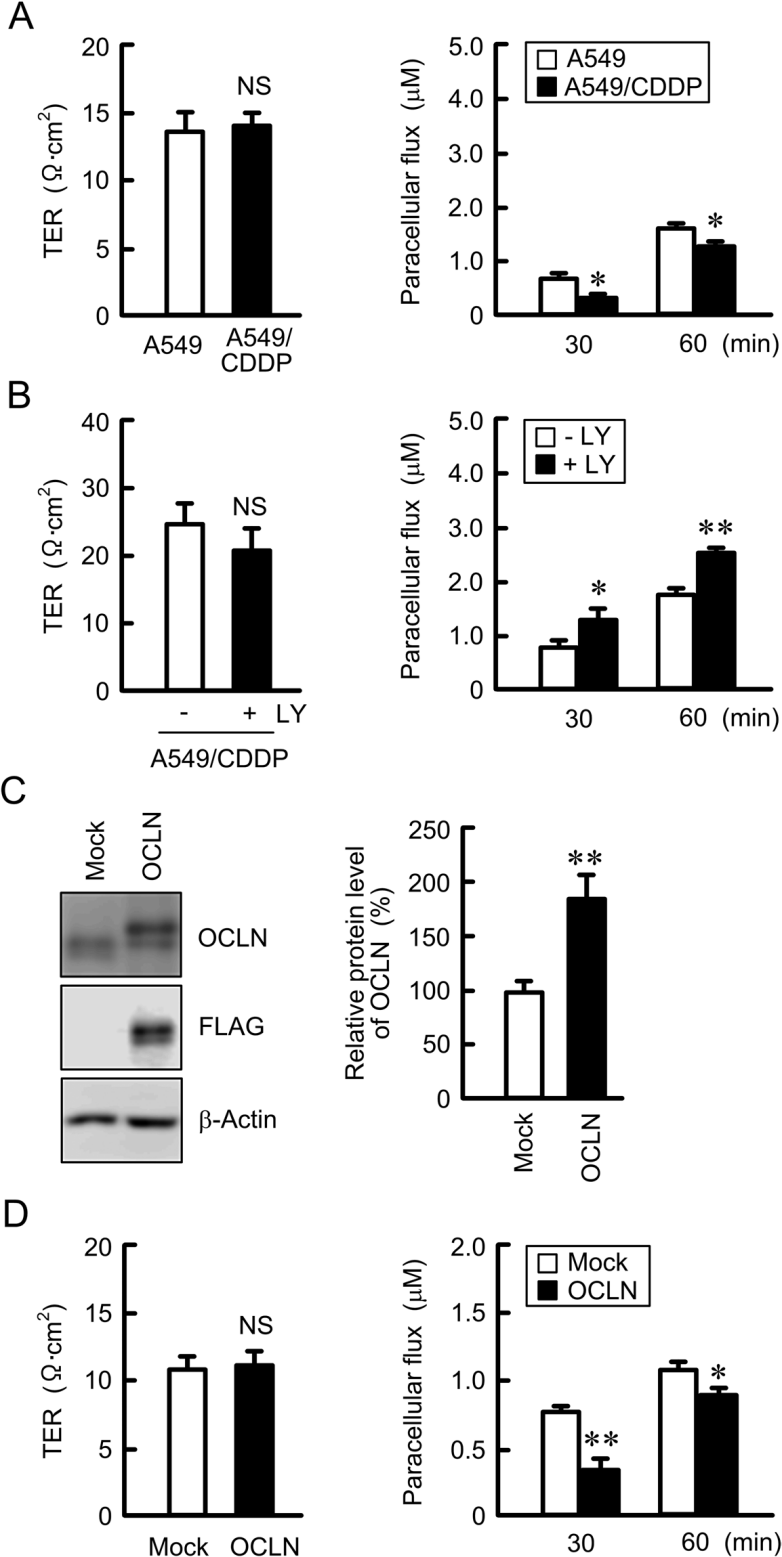
Effect of OCLN expression on tight junction permeability. (**A** and **B**) A549 and A549/CDDP cells were plated on transwell inserts. As indicated, A549/CDDP cells were treated with or without of 10 μM LY-294002 (LY) for 24 h. TER and paracellular DXR flux were analyzed by a volt ohmmeter and fluorescence spectrometry, respectively. (**C**) Cell lysates were prepared from empty (mock) or FLAG-tagged OCLN-expressing cells. The expression of OCLN, FLAG, and β-actin was examined by western blotting. The protein levels of OCLN are shown as a percentage of the values in mock cells. The full-length blot images are shown in Supplementary Fig. S3. (**D**) TER and the paracellular DXR flux were measured in mock and OCLN-expressing cells. n = 3–4. **P < 0.01 and *P < 0.05 compared with A549. NS, *P* > 0.05.

CDDP resistance did not change transepithelial electrical resistance (TER) in A549 cells, but inhibited the paracellular permeability to DXR (Fig. 3A). Similarly, LY-294002 did not change TER in A549/CDDP cells, but it enhanced paracellular DXR flux (Fig. 3B). To clarify the involvement of OCLN in the regulation of TJ properties, we established stably OCLN-expressing A549 cells (OCLN/A549). The expression of OCLN was confirmed by western blotting using anti-FLAG and anti-OCLN antibodies (Fig. 3C). The overexpression of OCLN inhibited paracellular DXR flux without affecting TER (Fig. 3D). These results were similar to those in A549/CDDP cells.

### Effect of OCLN overexpression on toxicity against anticancer drugs in A549 cells

**Figure 4:**
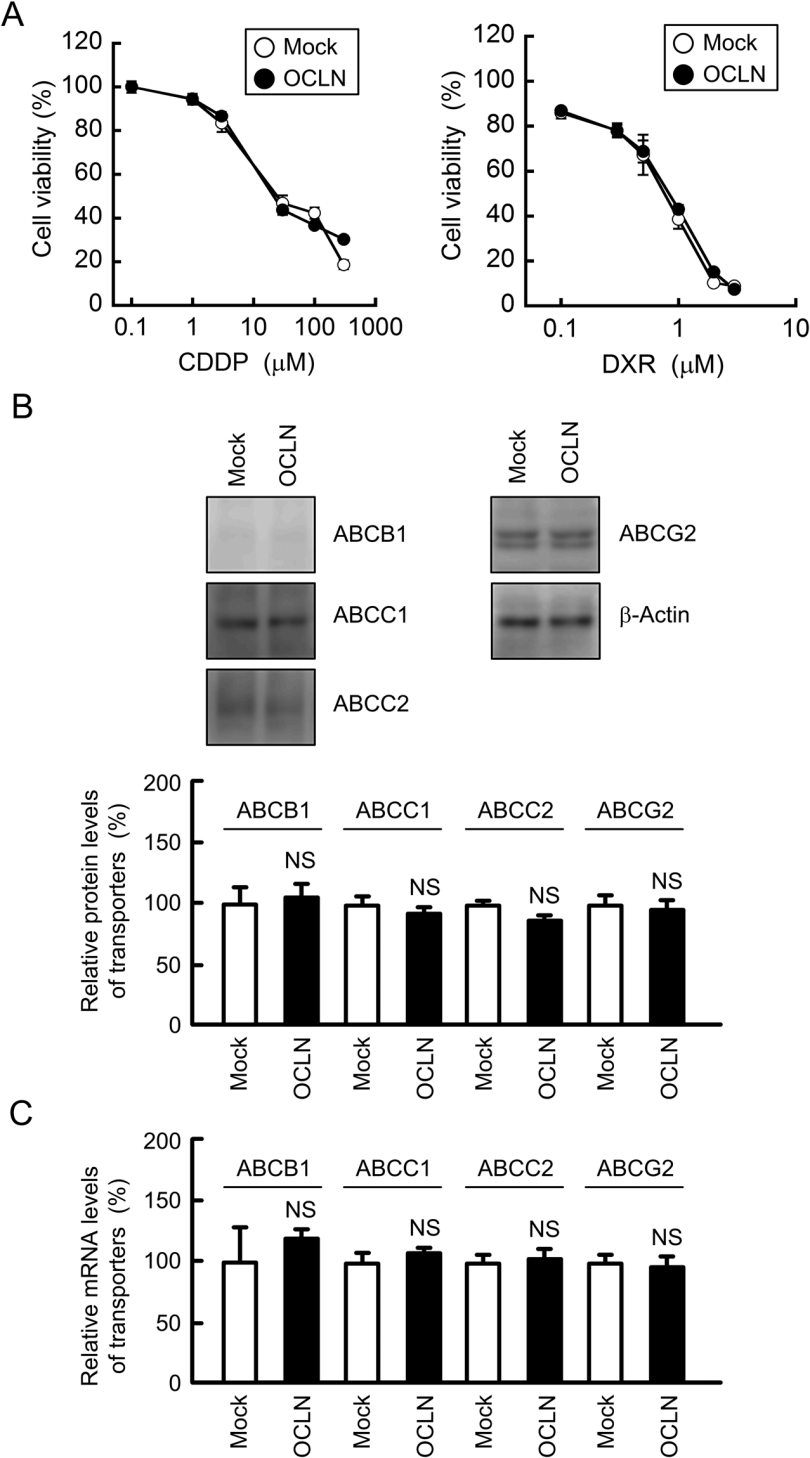
Effect of OCLN overexpression on cytotoxicity of anticancer drugs. (**A**) Mock and OCLN-expressing cells were treated with CDDP or DXR for 48 h. Cell viability was measured by WST-1 assays. (**B**) The expression of ABCB1, ABCC1, ABCC2, ABCG2, and β-actin in cell lysates was examined by western blotting. The protein levels of ABC transporters are shown as a percentage of the values in mock cells. The full-length blot images are shown in Supplementary Fig. S4. (**C**) The mRNA levels of ABCB1, ABCC1, ABCC2, ABCG2 are shown as a percentage of the values in mock cells. n = 3–6. NS, *P* > 0.05 compared with mock cells.

The toxicity against anticancer drugs was estimated in 2D monolayer culture of A549 cells. Both CDDP and DXR dose-dependently increased cytotoxicity (Fig. 4A). The overexpression of OCLN did not affect the toxicity against anticancer drugs. In western blotting and quantitative real time polymerase chain reaction (PCR) analyses, both protein and mRNA levels of ATP-binding cassette (ABC) transporters, including ABCB1, ABCC1, ABCC2, and ABCG2 were not changed by OCLN overexpression (Fig. 4B,C). These results indicated that OCLN may not be directly involved in the chemoresistance in 2D culture model.

### Effect of CDDP resistance on formation of spheroids

**Figure 5:**
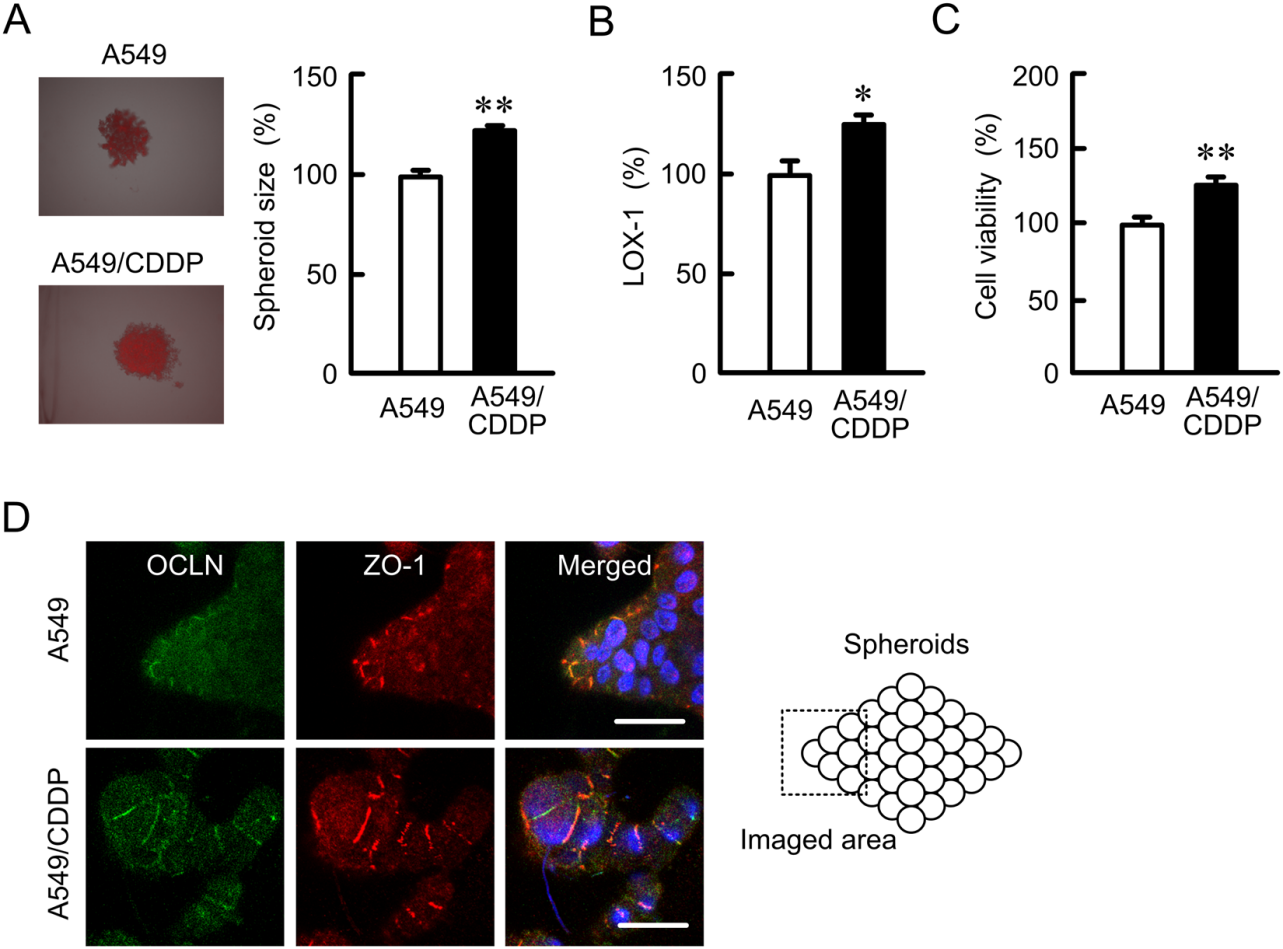
Elevation of hypoxic level and cell viability in spheroid model by CDDP resistance. (**A** and **B**) A549 and A549/CDDP cells were plated on PrimeSurface96U multi-well plates. After treating the cells with 2 μM LOX-1 for 24 h, the fluorescence images were acquired using a fluorescence microscope (left images). The spheroid size and fluorescence intensities of LOX-1 are represented as a percentage of the values in A549 cells. (**C**) The viability of spheroid cells was assessed by a CellTiter-Glo 3D Cell Viability Assay kit. (**D**) Immunofluorescence stainings with anti-OCLN (green) and anti-ZO-1 (red) antibodies were performed. The right images show the merged picture with DAPI (blue). Scale bar represents 10 µm. n = 4–6. **P < 0.01 and *P < 0.05 compared with A549.

A549 cells form spheroids by culturing in round-bottom 96-well plates. A549/CDDP cells showed larger size of spheroids than A549 cells (Fig. 5A). The hypoxic level and cell viability in A549/CDDP cells were higher than those in A549 cells (Fig. 5B,C). In immunofluorescence measurement of spheroid cells showed that both OCLN and ZO-1 were distributed in the cell-cell border area of most outside cells (Fig. 5D). These results indicated that CDDP resistance may enhance viability and hypotonic stress in spheroid cells.

### Effect of OCLN overexpression on formation of spheroids

**Figure 6:**
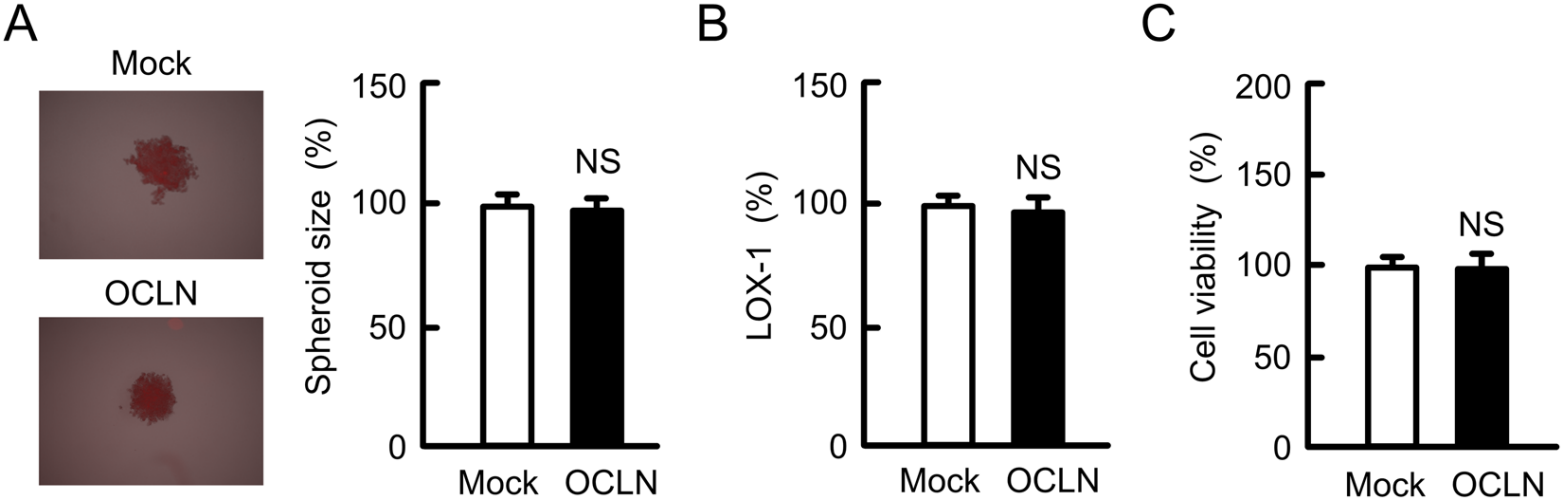
Effect of OCLN overexpression on hypoxic level and viability in spheroids. (**A** and **B**) Mock/A549, and OCLN/A549 cells were plated on PrimeSurface96U multi-well plates. After treating the cells with 2 μM LOX-1 for 24 h, the fluorescence images were acquired (left images). The spheroid size and fluorescence intensities of LOX-1 are represented as a percentage of the values in mock cells. (**C**) The viability of spheroid cells was measured. n = 4–6. NS, *P* > 0.05 compared with mock cells.

As described above, the size of spheroid in A549/CDDP cells was larger than that in A549 cells, but OCLN overexpression did not change the size (Fig. 6A). In addition, OCLN overexpression had no effect on hypoxic level (Fig. 6B) and cell viability (Fig. 6C). These results indicated that the phenomena of OCLN overexpressing cells are not incompletely matched with those of A549/CDDP cells. OCLN may not be directly implicated in the growth of spheroids.

### Elevation of chemoresistance spheroid cells by OCLN overexpression

**Figure 7:**
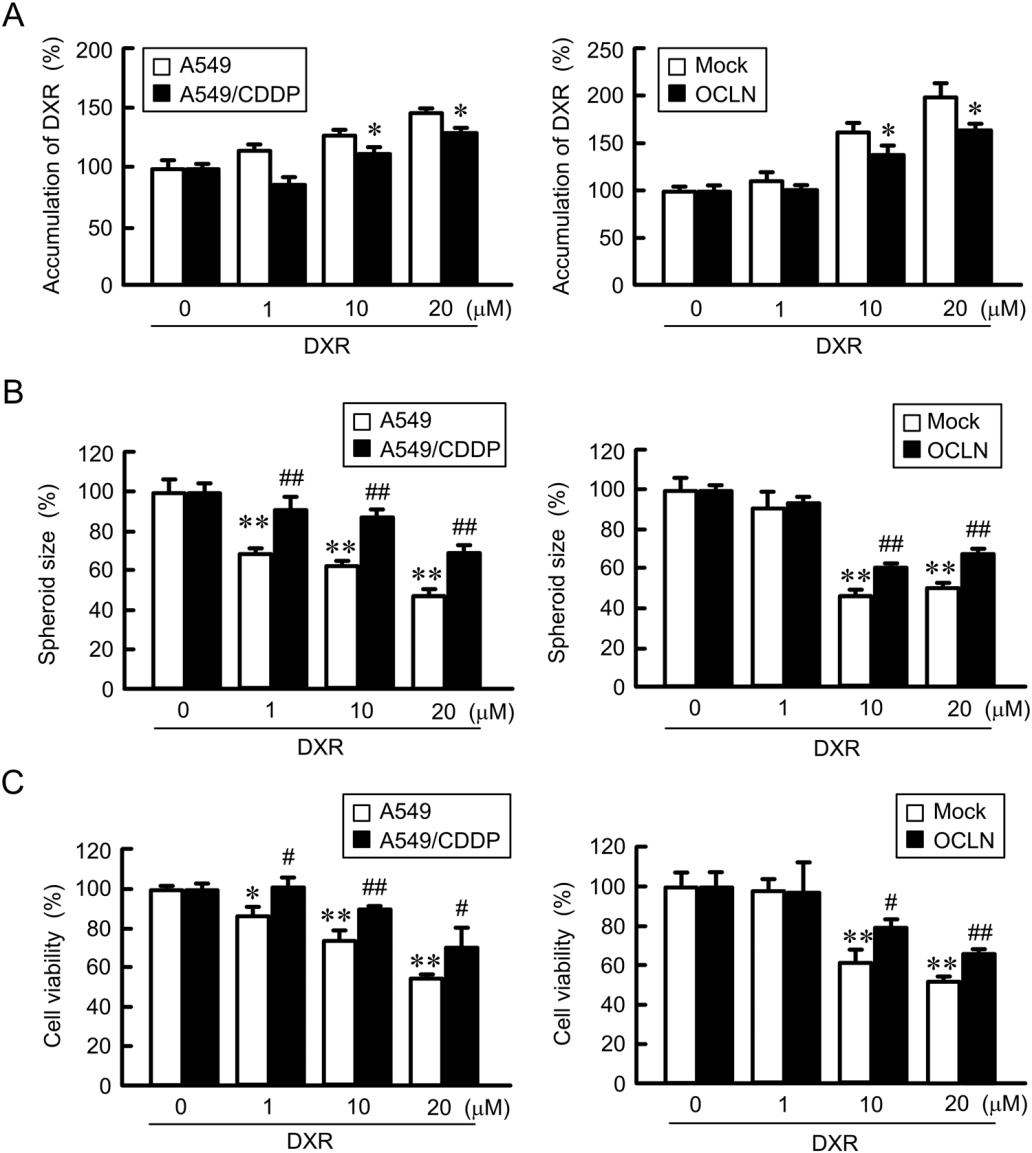
Decrease in DXR toxicity by OCLN overexpression in spheroid model. (**A**) A549, A549/CDDP, mock/A549, and OCLN/A549 cells were plated on PrimeSurface96U multi-well plates. The cells were incubated with DXR for 60 min. The fluorescence intensities of DXR in spheroids are shown as a percentage of 0 μM. (**B** and **C**) After treating the cells with DXR for 24 h, the spheroid size and viability of spheroid cells were measured. These values are represented as a percentage of 0 µM DXR. n = 3–4. **P < 0.01 and *P < 0.05 compared with 0 μM. ^**^P < 0.01 and ^*^P < 0.05 compared with 0 μM. ^##^*P* < 0.01 and ^#^*P* < 0.05 compared with A549 or mock.

The fluorescence intensity of DXR in the spheroids dose-dependently elevated in A549 cells, indicating that DXR was accumulated in the spheroids (Fig. 7A). The accumulation of DXR was significantly inhibited by CDDP resistance and OCLN overexpression. DXR dose-dependently decreased the size and viability in spheroid cells (Fig. 7B,C), which were recovered by CDDP resistance and OCLN overexpression. These results indicated that OCLN overexpression may enhance chemoresistance in spheroid cells.

### Effect of *OCLN* siRNA on paracellular permeability to DXR and cytotoxicity

**Figure 8:**
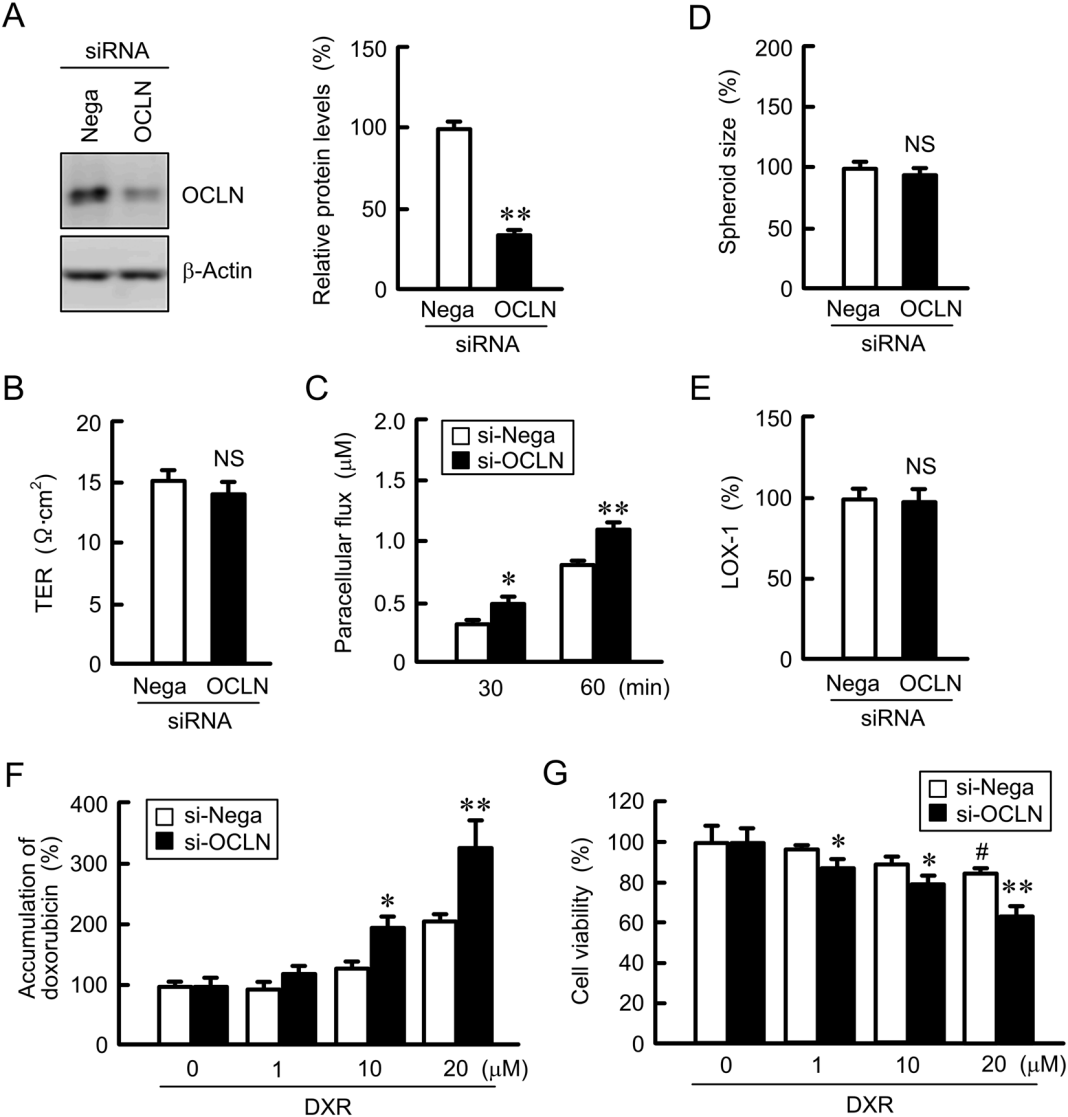
Increase in DXR toxicity by *OCLN* siRNA in spheroid model. (**A**) The expression of OCLN and β-actin in A549/CDDP cells transfected with negative (Nega) or *OCLN* siRNA were examined by western blotting. The protein levels of OCLN are shown as a percentage of the values in negative siRNA. The full-length blot images are shown in Supplementary Fig. S5. (**B**) TER was measured in negative and *OCLN* siRNA-transfected A549/CDDP cells. (**C**) Paracellular DXR flux for 30 and 60 min was measured in negative and *OCLN* siRNA-transfected A549/CDDP cells. (**D** and **E**) A549/CDDP cells transfected with negative or *OCLN* siRNA were plated on PrimeSurface96U multi-well plates. The spheroid size and fluorescence intensity of LOX-1 are represented as a percentage of the values in negative siRNA. (**F**) Negative and *OCLN* siRNA-transfected A549/CDDP cells were incubated with DXR for 60 min at the concentrations indicated. The fluorescence intensities of DXR in spheroids are shown as a percentage of 0 μM. (**G**) Negative and *OCLN* siRNA-transfected A549/CDDP cells were incubated with DXR for 24 h at the concentrations indicated. The viability of spheroid cells was assessed. n = 3–4. **P < 0.01 and *P < 0.05 compared with negative siRNA. ^##^*P* < 0.01 and ^#^*P* < 0.05 compared with 0 μM DXR. NS, *P* > 0.05 compared with negative siRNA.

Next, the effect of *OCLN* siRNA on paracellular DXR permeability was examined in 2D model and on DXR-induced chemoresistance in A549/CDDP spheroid cells. The protein level of OCLN was decreased over 60% by *OCLN* siRNA in A549/CDDP cells (Fig. 8A). The introduction of *OCLN* siRNA in A549/CDDP cells increased paracellular permeability to DXR (Fig. 8B). In contrast, TER was unchanged by OCLN knockdown (Fig. 8C). Neither spheroid size nor hypoxic level were changed by *OCLN* siRNA (Fig. 8D,E). The accumulation and toxicity of DXR in A549/CDDP cells were increased by *OCLN* siRNA (Fig. 8F,G). These results show an inverse relationship of OCLN expression with CDDP sensitivity, indicating that the increase in OCLN expression may be implicated in reducing chemosensitivity of A549/CDDP spheroid cells.

### Increase in CDDP and SN-38 toxicities of A549/CDDP spheroid cells by *OCLN* siRNA

**Figure 9:**
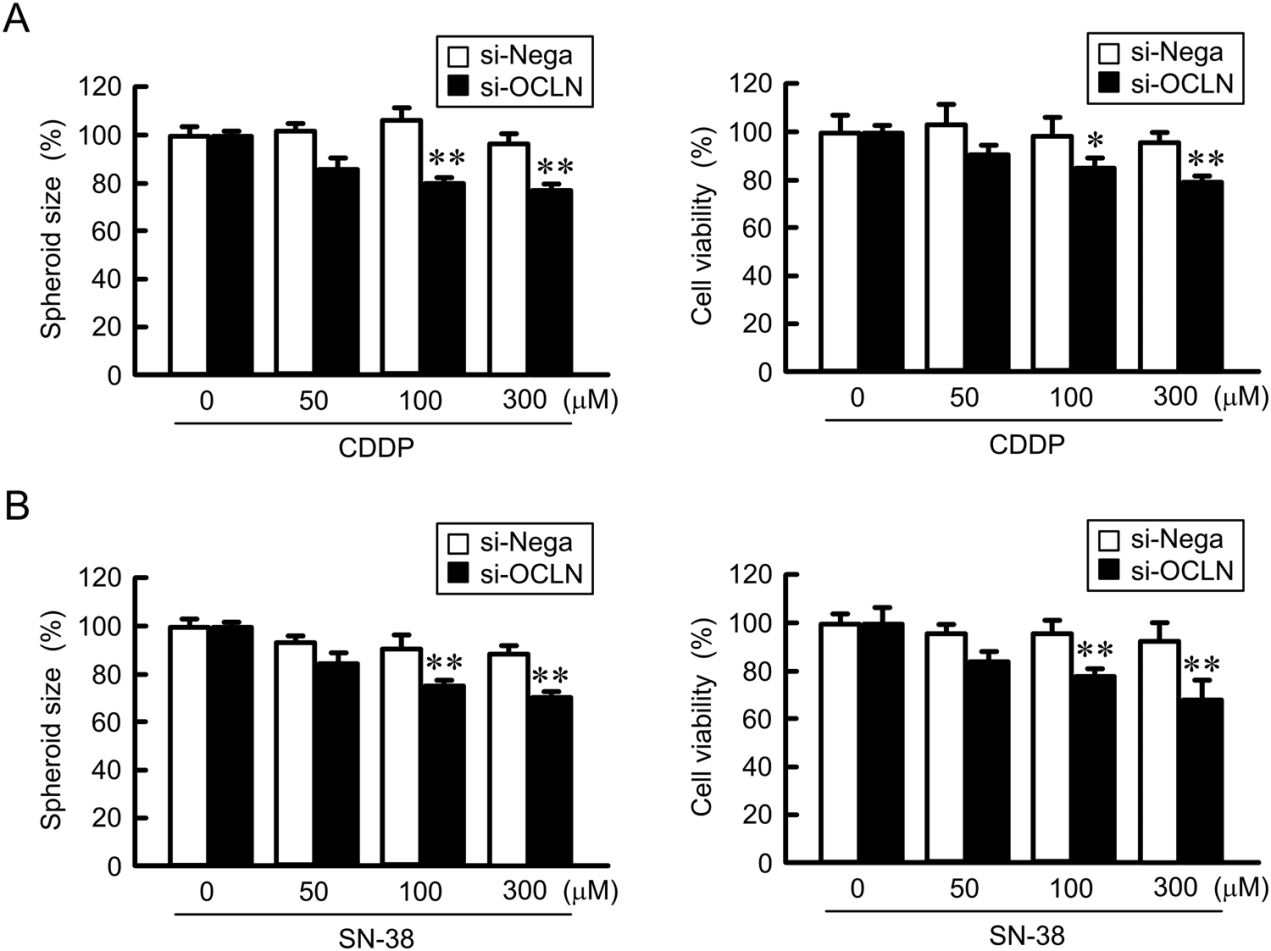
Enhancement of chemosensitivity by OCLN knockdown in A549/CDDP spheroid cells. Negative control or *OCLN* siRNAs were transfected in A549/CDDP cells. After 48 h of transfection, the cells were treated with CDDP (**A**) or SN-38 (**B**) for additional 48 h. The size and cell viability of spheroids are shown as a percentage of negative siRNA. n = 3–4. **P < 0.01 and *P < 0.05 compared with negative siRNA.

As shown above, *OCLN* siRNA significantly enhanced DXR toxicity in A549/CDDP cells. To clarify the effects of other anticancer drugs toxicities, we examined the effect of CDDP and SN-38 toxicities in A549/CDDP spheroid cells. Both CDDP and SN-38 dose-dependently decreased the spheroid size and cell viability, which were exaggerated by *OCLN* siRNA (Fig. 9). The effect of OCLN knockdown on CDDP and SN-38 toxicities agree with that on DXR toxicity. These results indicated that the elevation of OCLN expression in spheroids may cause resistance to lung cancer chemotherapy.

## Discussion

A549/CDDP cells acquired resistance to not only CDDP, but also DXR (Fig. 1A). Similarly, cross resistance to anticancer drugs are reported in docetaxel-resistant^23^ and gemcitabine-resistant A549 cells^24^. The detailed mechanism of acquisition of chemoresistance has not been fully understood. In the present study, we found for the first time that OCLN expression is elevated in anticancer drug-resistant A549 cells (Fig. 1). Similarly, the increase in OCLN mRNA was also observed in CDDP-resistant RERF-LC-MS and PC-3 cells, indicating that the induction of OCLN may commonly occur in chemoresistant lung adenocarcinoma cells. Furthermore, OCLN may affect the function of TJs because the induced OCLN is localized at the TJs.

So far, the activation of ERK1/2 and Akt pathways, two major cell survival pathways, has been reported in CDDP-resistant gastric cancer cells^25^ and ovarian cancer cells^26^. The inhibition of ERK1/2 or Akt pathway re-sensitizes the resistant cells to CDDP. Similarly, our data indicated that the levels of p-ERK1/2 and p-Akt in A549/CDDP cells are greater than those in normal A549 cells (Fig. 2A). The regulatory mechanism of OCLN expression is not fully understood, but it is upregulated by a JAK-STAT3 signaling pathway in colon SW480 and HCT116 cells^27^. The CDDP-resistance induced elevation of OCLN expression was inhibited by LY-294002, but not by U0126 (Fig. 2B,C), indicating that OCLN expression is increased by the activation of PI3K/Akt pathway in A549 cells. The upregulation mechanism of OCLN may be different in tissue types. There is a possibility that OCLN confers resistance to anticancer drugs because the expression of OCLN is regulated by a common signaling pathway with chemoresistance including ERK1/2 or Akt. However, the overexpression of OCLN in A549 cells had no effect on CDDP and DXR toxicities in 2D culture model (Fig. 4A). We recently found that CDDP resistance increases the expression level of ABCC2 in A549 cells (data not shown). In addition, the expression of ABCC2 is positively correlated with CLDN2 expression^8^. However, the overexpression of OCLN did not change the protein and mRNA levels of ABC transporters including ABCC2 (Fig. 4B,C). The overexpression of OCLN is suggested not to be directly implicated in the acquisition of chemoresistance in A549 cells.

The properties of paracellular permeability to larger molecules are also characterized by CLDNs. The activation of checkpoint kinase 1 decreases paracellular permeability to fluorescein isothiocyanate-dextran (MW: 4 kDa, FD-4) mediated via the elevation of CLDN5 in Caco-2 and T84 cells^28^. The knockdown of CLDN2 by siRNA decreases the paracellular flux of urea (MW: 60) in Caco-2 and renal tubular MDCK II cells^29^. We recently reported that paracellular permeability to lucifer yellow (LY, MW: 457), a tracer for paracellular transport, was increased by CLDN2 knockdown in A549 cells^8^. Few reports show that OCLN is also implicated in the regulation of paracellular transport of larger molecules. Knockdown of *OCLN* expression by siRNA increases transepithelial flux of urea (MW: 60), mannitol (MW: 182), inulin (MW: 5,000), and dextran (MW: 10,000 and 70,000) in Caco-2 cells^30^. Similarly, internalization of OCLN increases paracellular permeability to FD-4 in Caco-2 cells^31^ and LY in mouse MCE301 cells^32^. We found that OCLN overexpression significantly suppresses paracellular permeability to DXR (MW: 543) in A549 cells. OCLN may function as paracellular barrier to large molecules in chemoresistant lung adenocarcinoma cells.

Paracellular permeability to DXR was suppressed by CDDP-resistance, which were recovered by LY-294002 without affecting TER (Fig. 3A,B). These results suggest that OCLN may not be essential to regulate paracellular permeability to ions, but we cannot deny the involvement of other CLDNs. Actually, the expression of CLDN1 was also elevated by CDDP-resistance in A549 cells, which was blocked by LY-294002^9^. The balance of the expression of CLDNs and OCLN may induce this property of paracellular permeability to ions. However, our data (Fig. 3D) and other reports^33^ strongly support that OCLN is not implicated in the regulation of paracellular ion flux. Few reports demonstrate that TER is changed by a decrease in OCLN expression^34^, which may be indirectly caused by the change of CLDNs expression or interaction with an adaptor protein, ZO-1.

The microenvironment formed by tumor cells in the body could be a cause of drug resistance. The hypoxic level was increased in A549/CDDP cells compared to A549 cells (Fig. 5), suggesting that A549/CDDP cells are exposed to more severe hypoxic environment compared to A549 cells. In contrast, the hypoxic level and viability in A549/OCLN cells were similar to those in mock cells (Fig. 6). The correlation between junctional proteins and the formation of stress environments in spheroids are not fully understood. In immunofluorescence measurement of spheroid cells showed that both OCLN and ZO-1 are distributed in the cell-cell border area of most outside cells (Fig. 5D). We suggest that OCLN is not directly implicated in the formation of hypoxic environment in A549 spheroid cells. Conversely, the sensitivity against anticancer drugs was influenced by overexpression and knockdown of OCLN (Figs 7–9). These results suggest that OCLN enhances chemoresistance mediated through blocking of accumulation of anticancer drugs in spheroids.

Taken together, OCLN expression was elevated by CDDP resistance mediated through the activation of Akt in A549 cells. OCLN expression was also induced by resistance to DXR, SN-38, and GEM in A549 cells, and resistant to CDDP in RERF-LC-MS and PC-3 cells. The overexpression of OCLN inhibited the paracellular DXR flux in 2D monolayers, but did not change chemoresistance. In contrast, the anticancer drug-induced toxicities were enhanced by *OCLN* knockdown in A549/CDDP spheroid cells. OCLN may be not directly implicated in the acquisition of resistance to anticancer drugs in lung adenocarcinoma cells, but it suppresses chemosensitivity in spheroid cells. Our recent data indicate that CLDN1 and CLDN2 are also implicated in the development of chemoresistance in 3D spheroid cells^8,9^. These tight junctional proteins may be new targets of adjuvant chemotherapy in lung adenocarcinoma.

## Material and Methods

### Materials

Mouse anti-ZO-1 monoclonal antibody (33–9100, Lot: 1100420A) and Lipofectamine 2000 were obtained from Thermo Fisher Scientific (Rockford, IL, USA). Rabbit anti-p-Akt (4060, Lot: 14), anti-Akt (4691, Lot: 20), anti-p44/42 MAPK (ERK1/2, 4695, Lot: 1), anti-c-Fos (2250, Lot: 4), and anti-MRP2 (ABCC2, 4446, Lot: 1) polyclonal antibodies were from Cell Signaling Technology (Beverly, MA, USA). Goat anti-β-actin (sc-1615, Lot: C141), rabbit anti-p-ERK1/2 (sc-16982R, Lot: L0704) polyclonal antibodies and mouse anti-p-c-Fos (sc-81485, E2008) monoclonal antibodies were from Santa Cruz Biotechnology (Santa Cruz, CA, USA). Rabbit anti-ABCB1 (GTX108354, Lot: 39834), anti-ABCC1 (GTX116046, Lot: 40135), and anti-ABCG2 (GTX100437, Lot: 39471) polyclonal antibodies were from GeneTex (Irvine, CA, USA). Mouse anti-FLAG (018-22381, Lot: SAQ1191) monoclonal antibody, CDDP, and DXR were from Wako Pure Chemical (Osaka, Japan). LY-294002, and U0126 were purchased from BIOMOL Research Laboratories (Plymouth Meeting, PA, USA) and Sigma-Aldrich (Saint Louis, MO, USA), respectively. All other reagents were of the highest purity commercially available.

### Cell culture

A549 cells derived from human lung adenocarcinoma were obtained from the RIKEN BRC through the National Bio-Resource Project of the MEXT (Ibaraki, Japan). Human lung adenocarcinoma RERF-LC-MS (JCRB0081) and PC-3 (JCRB0077) cells were obtained from the JCRB Cell Bank (National Institute of Health Sciences, Tokyo, Japan). The cells were grown in Dulbecco’s modified Eagle’s medium (DMEM, Sigma-Aldrich) supplemented with 5% fetal calf serum (FCS, HyClone, Logan, UT, USA), 0.07 mg/ml penicillin-G potassium, and 0.14 mg/ml streptomycin sulfate in a 5% CO_2_ atmosphere at 37 °C.

### Plasmid DNA construction and transfection

Flag-tagged OCLN vector (#86042) was purchased from Addgene (Cambridge, MA, USA). The *OCLN* cDNA was subcloned into pTRE2-hyg vector (Clontech Laboratories, Mountain View, CA, USA). The siRNAs for negative control and *OCLN* were purchased from Santa Cruz and Sigma-Aldrich, respectively. Mock (empty vector), *OCLN*/pTRE2 vector, or siRNA was transfected into cells using Lipofectamine 2000 as recommended by the supplier. Stable transfectants of mock and OCLN were selected with 400 μg/ml hygromycin B and picked with a ring clone. Subsequently the cells were subsequently maintained in the presence of 50 μg/ml hygromycin B. To establish resistant cells against CDDP, DXR, SN-38, and GEM, A549 cells were continuously cultured in the growth medium supplemented with these anticancer drugs, whose concentration was increased in a stepwise manner (CDDP: 1, 2, 3, 4, and 5 μM; DXR: 1, 5, 10, 15, and 20 nM; SN-38: 10, 20, 30, 40, and 50 nM; GEM: 1, 2, 3, 4 and 5 nM) for 4–6 weeks. The established resistant cells were maintained in the growth medium containing 5 μM CDDP, 20 nM DXR, 50 nM SN-38, or 5 nM GEM. The cell growth was completely inhibited at higher concentration of anticancer drugs. Similarly we obtained CDDP-resistant RERF-LC-MS and PC-3 cells.

### 3D culture spheroid model

Cells were plated at densities of 1 × 10^4^ cells/well on PrimeSurface96U multi-well plates (Sumitomo Bakelite, Tokyo, Japan). After culturing for 96 h, the size and viability of spheroids were measured as described previously^9^. The fluorescence intensities of DXR and LOX-1, a hypoxia probe, were calculated using ImageJ software.

### Cytotoxicity of anticancer drugs

Cells were plated on 96-well flat bottomed plates or PrimeSurface96U multi-well plates. Anticancer drugs were applied for 24 h in FCS-free media. In 2D and 3D culture models, the cell viability was measured using a Premix WST-1 Cell Proliferation Assay Kit (Takara, Otsu, Japan) and a CellTiter-Glo 3D Cell Viability Assay kit (Promega, Madison, WI, USA), respectively.

### Isolation of total RNA and quantitative real time PCR

Total RNA was extracted using TRI reagent (Sigma-Aldrich). Reverse transcription and quantitative real time PCR was performed as described previously^35^. The primer pairs used for PCR are listed in Table 1.

**Table 1:** Primers for real time PCR.

Genes	Direction	Sequence
*OCLN*	Sense	5′-TTTGTGGGACAAGGAACACA-3′
*OCLN*	Antisense	5′-TCATTCACTTTGCCATTGGA-3′
*ABCB1*	Sense	5′-CCCATCATTGCAATAGCAGG-3′
*ABCB1*	Antisense	5′-TGTTCAAACTTCTGCTCCTGA-3′
*ABCC1*	Sense	5′-ATGTCACGTGGAATACCAGC-3′
*ABCC1*	Antisense	5′-GAAGACTGAACTCCCTTCCT-3′
*ABCC2*	Sense	5′-ACAGAGGCTGGTGGCAACC-3′
*ABCC2*	Antisense	5′-ACCATTACCTTGTCACTGTCCATGA-3′
*ABCG2*	Sense	5′-AGATGGGTTTCCAAGCGTTCAT-3′
*ABCG2*	Antisense	5′-CCAGTCCCAGTACGACTGTGACA-3′
*β-Actin*	Sense	5′-CCTGAGGCACTCTTCCAGCCTT-3′
*β-Actin*	Antisense	5′-TGCGGATGTCCACGTCACACTTC-3′

### Sodium dodecyl sulfate (SDS)-polyacrylamide gel electrophoresis and western blotting

Confluent A549 cells were scraped into cold phosphate-buffered saline and precipitated by centrifugation. Then, the cells were lysed in a RIPA buffer (150 mM NaCl, 50 mM Tris-HCl (pH8.0), 1% Triton X-100, 0.1% SDS, 0.5 mM EDTA) supplemented with a protease inhibitor cocktail (Sigma-Aldrich), and sonicated for 20 s. The lysates were used as whole cell extracts. After centrifugation at 6,000 x g for 5 min, the resultant supernatants which include membrane and cytoplasmic proteins were used as cell lysates. SDS-polyacrylamide gel electrophoresis and western blotting were carried out as described previously^9^.

### Confocal microscopy

Cells were plated on cover glasses. After forming confluent monolayer, the cells were incubated with 4% paraformaldehyde for 15 min at 4 °C and then permeabilized with 0.2% Triton X-100 for 15 min. Following permeabilization, the cells were blocked with 4% Block Ace (Dainippon Sumitomo Pharma, Osaka, Japan) for 30 min and incubated with anti-OCLN (1:100 dilution) and anti-ZO-1 (1:100 dilution) antibodies for 16 h at 4 °C. They were then incubated with Alexa Fluor 488- and 555-conjugated antibodies (1:100 dilution) for 1.5 h at room temperature. In 3D culture model, the cells plated on PrimeSurface96U multi-well plates were cultured for 96 h. Following incubation with 4% paraformaldehyde for 30 min, the cells were permeabilized with 0.2% Triton X-100 for 30 min. After blocking with Block Ace, they were stained with anti-OCLN (1:100 dilution) and anti-ZO-1 (1:100 dilution) antibodies for 16 h at 4 °C, followed by incubation with secondary antibodies as described above. For transparency, the samples were treated with Visikol-HISTO-M (Visikol, Whitehouse Station, NJ, USA). The fluorescence images were observed using an LSM 700 confocal microscope (Carl Zeiss, Jena, Germany).

### Measurement of paracellular permeability

Cells were plated at densities of 5 × 10^4^ cells on transwell plates (0.4 μm pore size, Corning Incorporated, Corning, NY, USA). After forming confluent monolayer, TER and paracellular permeability to DXR were measured as described previously^9^.

### Statistical analysis

All experiments were performed using at least 3 independent samples. Data are presented as means ± standard errors of mean. Analysis between two groups was done using Student’s *t* test. For comparison between multiple groups, one-way analysis (ANOVA) followed by Tukey’s multiple comparison test was used. Statistical analysis was performed with KaleidaGraph version 4.5.1 software (Synergy Software, PA, USA). *p* value < 0.05 was considered to be statistically significant.

## Electronic supplementary material

Dataset 1
