# Supplementary material for: Increase in resistance to anticancer drugs involves occludin in spheroid culture model of lung adenocarcinoma A549 cells
Source: Sci Rep. 2018 Oct 11;8:15157. doi: 10.1038/s41598-018-33566-w (PMC6181945; doi:10.1038/s41598-018-33566-w)
Supplement: Supplementary file 1 — Dataset 1 [file 41598_2018_33566_MOESM1_ESM.doc]

Supplementary Information

Increase in resistance to anticancer drugs involves occludin

in spheroid culture model of human lung adenocarcinoma A549 cells

**Hiroaki Eguchi^1^, Risa Akizuki^1^, Ryohei Maruhashi^1^, Mitsutoshi Tsukimoto^2^,**

**Takumi Furuta^3^, Toshiyuki Matsunaga^1^, Satoshi Endo^1^, and Akira Ikari^1^**

**^1^ From the Laboratory of Biochemistry, Department of Biopharmaceutical Sciences,**

**Gifu Pharmaceutical University, Japan**

**^2^ Department of Radiation Biosciences, Faculty of Pharmaceutical Sciences,**

**Tokyo University of Science, Japan**

**^3^ Department of Pharmaceutical Chemistry, Kyoto Pharmaceutical University, Japan**

To whom correspondence should be addressed: Akira Ikari, Ph.D.

Laboratory of Biochemistry,

Department of Biopharmaceutical Sciences,

Gifu Pharmaceutical University,

1-25-4 Daigaku-nishi, Gifu 501-1196, Japan,

Tel: +81-58-230-8124; Fax: +81-58-230-8124

E-mail: ikari@gifu-pu.ac.jp

**Supplementary Figure S1.** Full images of the blots from figure 1B.

**Supplementary Figure S2.** Full images of the blots from figure 2A and 2B.

**Supplementary Figure S3.** Full images of the blots from figure 3C.

**Supplementary Figure S4.** Full images of the blots from figure 4B.

**Supplementary Figure S5.** Full images of the blots from figure 8A.
